# Sequential therapy for hereditary leiomyomatosis and renal cell cancer-associated renal cell carcinoma: a case report and report of a new family pedigree

**DOI:** 10.1093/omcr/omae060

**Published:** 2024-08-23

**Authors:** Ichiro Tsuboi, Momoko Araki, Shuhei Yokoyama, Gen Tanaka, Kazutaka Mitani, Saori Yosioka, Yusuke Kobayashi, Hirochika Nakajima, Taichi Nagami, Kohei Ogawa, Chiaki Koike, Koichiro Wada

**Affiliations:** Department of Urology, Shimane University Faculty of Medicine, Izumo, Japan; Department of Clinical Genetics Unit, Shimane University Faculty of Medicine, Izumo, Japan; Department of Urology, Shimane University Faculty of Medicine, Izumo, Japan; Department of Urology, Shimane University Faculty of Medicine, Izumo, Japan; Department of Urology, Shimane University Faculty of Medicine, Izumo, Japan; Department of Urology, Shimane University Faculty of Medicine, Izumo, Japan; Department of Urology, Shimane University Faculty of Medicine, Izumo, Japan; Department of Urology, Shimane University Faculty of Medicine, Izumo, Japan; Department of Urology, Shimane University Faculty of Medicine, Izumo, Japan; Department of Urology, Shimane University Faculty of Medicine, Izumo, Japan; Department of Urology, Shimane University Faculty of Medicine, Izumo, Japan; Department of Urology, Shimane University Faculty of Medicine, Izumo, Japan

**Keywords:** FoundationOne Liquid CDx, fumarate hydratase, hereditary leiomyomatosis and renal cell cancer, immune checkpoint inhibitor

## Abstract

Hereditary leiomyomatosis and renal cell cancer (HLRCC) is a rare autosomal-dominant disorder caused by a heterozygous germline mutation in the fumarate hydratase (*FH*) gene. HLRCC is clinically characterized by the development of three tumors: uterine leiomyomata, cutaneous leiomyomata, and renal cell carcinoma (RCC). HLRCC-associated RCC is aggressive and diagnosed at a much earlier age than sporadic RCC. It is essential for carriers of HLRCC to undergo annual renal screening by magnetic resonance imaging to detect early stage RCCs. Metastatic HLRCC-associated RCC must be treated by systemic therapy; however, it is unclear which medicines are most effective in treating this cancer owing to its low incidence rate. Immune checkpoint inhibitor (ICI) combinations or ICIs plus tyrosine kinase inhibitors are administered as systemic therapy for clear cell RCC. Here, we report a patient with HLRCC-associated RCC treated with sequential therapy, including ipilimumab plus nivolumab combination and cabozantinib, after diagnosis of HLRCC-associated RCC using FoundationOne Liquid CDx and single-site analysis. We also investigated familial *FH* mutations and describe a new family pedigree for HLRCC.

## Teaser

HLRCC is an autosomal-dominant disorder. The optimal therapeutic approach for metastatic HLRCC-associated renal cell carcinoma remains uncertain. It is imperative to examine blood relatives for the early identification and intervention of the condition.

## Introduction

Hereditary leiomyomatosis and renal cell cancer (HLRCC; OMIN #150800) is a rare autosomal-dominant disorder caused by a heterozygous germline mutation in the fumarate hydratase (*FH*) gene, which encodes an enzyme that is part of the tricarboxylic acid cycle [1]. A previous study estimated the prevalence of HLRCC to be 10 in 2 000 000 people [2]. HLRCC is clinically characterized by the development of three types of tumors: uterine leiomyomata (UL), cutaneous piloleiomyomata (CL) and renal cell carcinoma (RCC). The rates of UL and CL in at-risk individuals can be as high as 70%, whereas the rate of RCC in at-risk individuals is approximately 15%–20% [1, 2].

It is unclear why HLRCC-associated RCC is so aggressive. However, this characteristic of HLRCC-associated RCC has been recognized by clinicians. The mean age at diagnosis is in the early forties, indicating that this disease occurs much earlier than sporadic RCC; the youngest patient diagnosed with HLRCC-associated RCC was 7 years old [3]. Similar to sporadic RCC, the mean survival of individuals diagnosed with stage III/IV RCC is significantly shorter than that of individuals diagnosed with stage I/II RCC [2]. Therefore, it is essential for carriers of HLRCC to undergo renal screening annually with magnetic resonance imaging (MRI) to detect early stage RCC [1].

However, it is clinically difficult to detect nonmetastatic HLRCC-associated RCC owing to its aggressive nature. Although metastatic HLRCC-associated RCC should be treated with systemic therapy, the most effective medicines have not yet been identified due to the low incidence rate of this cancer. Currently, immune checkpoint inhibitor (ICI) combinations or ICIs plus tyrosine kinase inhibitors (TKIs) are administered as systemic therapies to treat clear cell RCC. Therefore, HLRCC-associated RCC has been treated with ICI combinations or ICIs plus TKIs [4–8].

Here, we report a patient with HLRCC-associated RCC treated with sequential therapy, including ipilimumab (IPI) plus nivolumab (NIVO) combination and cabozantinib (CABO), after diagnosis using FoundationOne Liquid CDx and single-site analysis. We also investigated familial *FH* mutations by single-site analysis, and we present a new family pedigree for HLRCC.

## Case presentation

**Figure 1:**
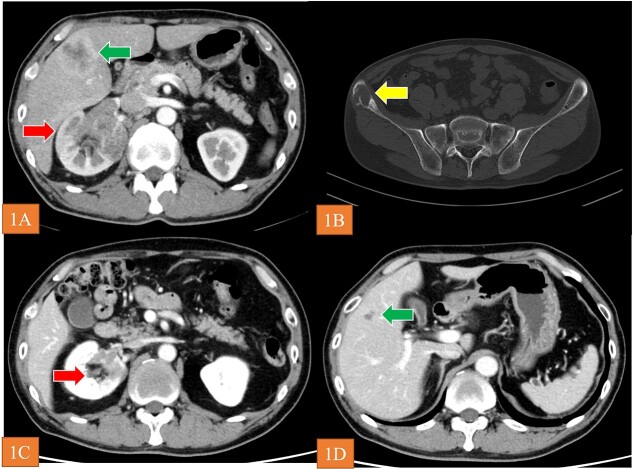
(A and B) CT revealed the renal tumor (arrow), liver tumor (arrow), and bone metastasis (arrow). (C and D) arrows show that the tumor volume decreased by 46% at 40 weeks after initiation of CABO.

A 56-year-old man visited a previous hospital due to an 8-month history of back pain. Contrast-enhanced computed tomography revealed a hypovascular solid mass in the right kidney and right lobe of the liver as well as osteolysis in the right ilium and lumbar spine at L4 (Fig. 1A and B). He was referred to shimane university hospital (Shimane, Japan) for further evaluation and treatment. His medical history was unremarkable. Blood tests revealed low hemoglobin level (10.7 g/dl), high blood platelet level (55.8 × 10^4^/μl), high C-related protein level (12.86 mg/dl), normal serum calcium level, and normal neutrophil counts. Urine test showed no microhematuria. The Karnofsky performance status was 90%. We then performed needle biopsy of the tumor in the right lobe of the liver. Histopathological findings for the specimen revealed unclassified RCC. There was no metastasis to the brain according to MRI. Accordingly, we diagnosed the patient with RCC at TNM stage cT3aN1M1. Based on the International Metastatic RCC Database Consortium risk classification, his risk was classified as poor. We selected IPI plus NIVO combination as the first-line systemic therapy. Because the patient was diagnosed with grade 1 myocarditis (CTCAE Version 5.0) after two courses of treatment, we halted administration of IPI plus NIVO. Instead, we started CABO 40 mg/day as second-line systematic therapy. The tumor volume decreased by 46% at 40 weeks (Fig. 1C and D), and the best overall response of CABO was a partial response within 73 weeks (RECIST Version 1.1). When disease progression was observed during CABO treatment, we prescribed axitinib 10 mg/day as third-line therapy. Utilizing FoundationOne Liquid on peripheral blood samples, we conducted an analysis of genetic mutations to aid in determining a more efficacious treatment approach. This analysis revealed a germline *FH* mutation (NM_000143.4) c.1189G>A (p.Gly397Arg) and we diagnosed as HLRCC-associated RCC. After AXI was no longer effective, we administered NIVO alone as fourth-line therapy. The patient is still alive as of March, 2023 (27 months after the start of treatment).

**Figure 2:**
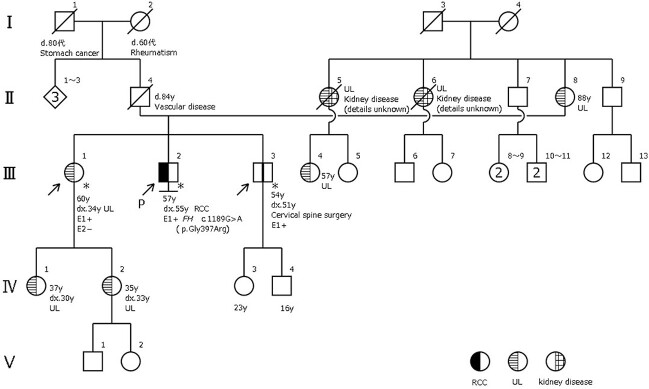
The index patient is identified by an arrow and the letter P. *Patients with FH mutations. RCC: renal cell carcinoma UL: uterine leiomyomata.

Because his older sister (60 years old) and younger brother (54 years old) wanted to know whether they had germline *FH* mutations, we performed single-site gene mutation analysis. Both siblings had *FH* mutations. Consequently, a family pedigree was established. Figure 2 shows a 5-generation family tree based on interviews with the patient and their family. III-2 is the index case. *FH* mutations were found in III-1 and III-3, who are his siblings. Furthermore, UL and kidney disease were both identified in the maternal aunts of the patients (II-5, 6), although the specific details are unknown. IV-1 and IV-2, who are the children of III-1, were diagnosed with UL at 30 and 35 years old, respectively, but have not yet undergone *FH* testing (Fig. 2). Hence, we will perform abdominal MRI for these *FH* mutation carriers annually to screen for renal cancer.

## Discussion

In this case report, we described a patient with HLRCC-associated RCC, which was detected by FoundationOne Liquid and single-site analysis, treated with IPI plus NIVO combination, CABO, AXI, and NIVO. Additionally, we assembled a pedigree delineating the tumor and clinical characteristics associated with HLRCC. In Japan, there have been 14 families with this cancer reported from 2007 to 2023 [3, 9, 10]. Given that the prevalence of HLRCC is approximately 10 in 2 000 000 people, it is estimated that there are approximately 600 carriers of HLRCC in Japan.

The optimal systemic therapy for metastatic HLRCC-associated RCC has not yet been identified owing to the low incidence rate of this cancer. In our case, we selected ICI combination therapy as the first-line treatment. A few case reports have demonstrated the efficacy of ICI and/or TKI treatment [5–7]. However, Xu et al. recently reported the ICI plus TKI combination therapy was associated with favorable overall survival in a multicenter retrospective study of 77 cases of *FH*-deficient RCC, including HLRCC [4]. Conventionally, although histopathological findings of HLRCC-associated RCC include papillary RCC, in practice, many histological types of RCC have been reported [2]. In the future, although further studies are needed to clarify the optimal therapy for HLRCC-associated RCC, systemic therapy focused on the histopathology or germline mutations of the disease, such as poly ADP (ribose) polymerase inhibitors, will be selected.

The *FH* mutation in this case was thought to have been inherited from the patient’s mother because there was a high incidence of UL on the maternal side and because the patient’s maternal aunts also had kidney disease. Therefore, screening for the early detection and treatment of renal cancer may be necessary for the children of the patient’s maternal aunts as well. In the future, we would like to proactively contact such families, provide information, and perform preventive screening if possible. When *FH* mutations are identified, it is important to reach a definitive diagnosis and to thoroughly investigate blood relatives from the perspective of preventive medicine as part of the treatment of hereditary tumors.

In conclusion, we report a patient with HLRCC-associated RCC treated with IPI plus NIVO combination, CABO, AXI, and rechallenge NIVO. We also created a new family tree for *FH* mutations.

## Conflict of interest

The authors declare no conflicts of interest.

## Approval of the research protocol by the Institutional Review Board (IRB)

This case report was approved by the IRB of Shimane University Hospital (approval no. 6892).

## Consent

Written informed consent was obtained from patient.
